# Artificial neural network identified the significant genes to distinguish Idiopathic pulmonary fibrosis

**DOI:** 10.1038/s41598-023-28536-w

**Published:** 2023-01-21

**Authors:** Zhongzheng Li, Shenghui Wang, Huabin Zhao, Peishuo Yan, Hongmei Yuan, Mengxia Zhao, Ruyan Wan, Guoying Yu, Lan Wang

**Affiliations:** State Key Laboratory of Cell Differentiation and Regulation, Henan International Joint Laboratory of Pulmonary Fibrosis, Henan Center for Outstanding Overseas Scientists of Pulmonary Fibrosis, College of Life Science, Henan Normal University, 46 Jianshe Road, Xinxiang, 453007 Henan China

**Keywords:** Computational biology and bioinformatics, Data mining, Machine learning

## Abstract

Idiopathic pulmonary fibrosis (IPF) is a progressive interstitial lung disease that causes irreversible damage to lung tissue characterized by excessive deposition of extracellular matrix (ECM) and remodeling of lung parenchyma. The current diagnosis of IPF is complex and usually completed by a multidisciplinary team including clinicians, radiologists and pathologists they work together and make decision for an effective treatment, it is imperative to introduce novel practical methods for IPF diagnosis. This study provided a new diagnostic model of idiopathic pulmonary fibrosis based on machine learning. Six genes including CDH3, DIO2, ADAMTS14, HS6ST2, IL13RA2, and IGFL2 were identified based on the differentially expressed genes in IPF patients compare to healthy subjects through a random forest classifier with the existing gene expression databases. An artificial neural network model was constructed for IPF diagnosis based these genes, and this model was validated by the distinctive public datasets with a satisfactory diagnostic accuracy. These six genes identified were significant correlated with lung function, and among them, CDH3 and DIO2 were further determined to be significantly associated with the survival. Putting together, artificial neural network model identified the significant genes to distinguish idiopathic pulmonary fibrosis from healthy people and it is potential for molecular diagnosis of IPF.

## Introduction

IPF is a chronic progressive interstitial lung disease characterized by developing lung scarring and deterioration of lung function^1^. The abnormal extracellular matrix accumulates with the lung tissue and vascular system are repeatedly micro-damaged, and the alveolar structure is destroyed, resulting in a decrease in the lung tissue's ability to exchange gas with the outside^2–4^. The clinical manifestations are cough and dyspnea, severe cases can lead to respiratory failure. IPF mainly occurs in people at 50 years of age and older, and the incidence increases with age^5–7^. IPF is widely believed to result from the interaction of genetics, environmental risk, and ageing^8^. Most IPF patients also have multiple complications, such as heart failure, gastroesophageal reflux disease, obstructive apnea, etc.^9,10^

The challenge to clinicians is distinguishing IPF from other idiopathic interstitial pneumonias, high-resolution CT of the chest and lung biopsy are advised now, but the risk of surgical lung biopsy is greater for elder pattens^11^. With the development of high-throughput sequencing technology, the molecular alteration can be observed and the changes at RNA level can be more accurately determined in disease^12–14^.

In addition, with the development of artificial intelligence technology, machine learning and deep learning models have recently made significant contributions to the development of predictive medicine and modern pathological practice^15–17^. These models enable people to better interpret high-throughput data, reduce the dependence of disease diagnosis on subjective consciousness, and provide more precise criteria for disease diagnosis^18–20^. In this study, Gene Expression Ontology (GEO) databases were used to screen the key genes and construct an artificial neural network model for diagnosis of idiopathic pulmonary fibrosis.

## Materials and methods

### Statement

Our study is based on open-source databases(GEO), there are no ethical issues and other conflicts of interest. which belongs to public databases. The patients involved in the database have been obtained ethical approval. Users can download relevant data for free for research and publish relevant articles.

### Data download and processing

We used keywords “idiopathic pulmonary fibrosis”, “pulmonary fibrosis”, and “lung fibrosis” to search for relevant datasets in the GEO database. Specifically, we included datasets in our analysis if they met the following criteria: (1) Adequate sample size. (2) Included both normal and disease groups. (3) RNA was extracted from lung tissue. (4) Adequate evidence for a diagnosis of IPF, such as HRCT. (5) Clinical information was collected in a standardized manner. In GSE47460, these are 582 total subjects, 254 have interstitial lung disease, 220 have COPD, and 108 are controls^21^. GSE110147 lung samples were obtained from the recipients’ organs of 22 patients with IPF, 10 with NSIP (non-specific interstitial pneumonia) and 5 with mixed IPF-NSIP undergoing lung transplantation^22^. In GSE53845 RNA was extracted directly from lung tissue samples from 40 IPF patients or 8 healthy controls^23^. In GSE70866, BAL cells were harvested from a discovery cohort of 62 patients from Freiburg, Germany, and two independent validation cohorts, Siena, Italy (50 patients) and Leuven, Belgium (64 patients)^24^. The GEOquery package was used to obtain the expression profiles and clinical phenotype data of the microarray datasets GSE47460, GSE53845, GSE110147, GSE32537, and GSE70866. Only IPF samples and normal samples for subsequent analysis were retained, excluding other lung samples. The annotation information of the chip probes of the corresponding platforms was obtained from the GEO database, respectively. During the conversion of the microarray probe ID and gene symbols, multiple probes corresponding to one gene symbol were found. Considering the reliability of the data, 'many-to-one' probe expression levels were used for gene expression levels. GSE47460 database had the largest sample size among the available databases and therefore provided the most possibility to identify differentially expressed genes.

### Differential gene expression and enrichment analysis

Principal Component Analysis (PCA) was performed to identify spatial sample separation in the patient cohort using an R package factoextra^25^. The R software package limma^26^ was used to achieved differential analysis on 91 control and 122 IPF samples of GSE47460, genes differentially expressed with *P* values < 0.05 and fold changes > 1.5 or < 2/3 were visualized using an R package heatmap. The metascape tool^27^ was used to carry out enrichment analysis with DEGs on multiple databases including GO database, KEGG database, Reactome database, Wikipathway database^28,29^.

### Random forest screens the top signatures

126 DEGs from GSE47460 (93 up-regulated and 33 down-regulated) were used to construct the random forest model (Table 1). The construction of random forest model and the chosen of top signatures were used the methods of Tian^1^. The R package pheatmap was used to perform k-means unsupervised clustering of the GSE47460 dataset and visualize.

**Table 1.** 126 DEGs from GSE47460.

logFC	AveExpr	t	*P* value	Adj. *P*.Val	B	Sig	Gene_name
2.766981	10.02813	24.83617	1.59E−71	2.41E−67	152.2407	Up	CDH3
3.51672	8.149571	24.37658	5.15E−70	3.91E−66	148.795	Up	IL13RA2
4.22487	4.938856	22.84845	6.52E−65	3.30E−61	137.1561	Up	IGFL2
3.364436	10.60868	22.74715	1.43E−64	5.44E−61	136.375	Up	COMP
4.365315	7.774525	22.39132	2.30E−63	6.99E−60	133.6222	Up	COL17A1
− 2.23625	7.195647	− 21.9088	1.02E−61	2.58E−58	129.8669	Down	CTNND2
2.991574	8.057767	21.5496	1.73E−60	3.76E−57	127.0559	Up	HS6ST2
2.760018	8.75285	21.38315	6.48E−60	1.23E−56	125.7486	Up	DIO2
2.487672	8.386943	20.96768	1.76E−58	2.97E−55	122.4737	Up	ADAMTS14
2.657568	6.986265	20.47869	8.76E−57	1.33E−53	118.5982	Up	SCG5
− 2.17148	6.87585	− 20.4486	1.12E−56	1.54E−53	118.3591	Down	FAM167A
2.976165	6.119909	19.82738	1.65E−54	1.79E−51	113.4033	Up	TMEM229A
− 2.06299	9.50816	− 19.8031	2.00E−54	2.03E−51	113.209	Down	CRTAC1
− 2.12757	10.86437	− 19.6535	6.71E−54	6.36E−51	112.0103	Down	C11orf9
2.87141	8.609686	18.7457	1.06E−50	7.31E−48	104.7026	Up	TUBB3
2.362009	4.947451	18.45088	1.17E−49	7.41E−47	102.3176	Up	FRMD5
2.572854	7.592293	18.30919	3.72E−49	2.17E−46	101.1696	Up	TDO2
2.127251	10.95033	17.93908	7.67E−48	3.33E−45	98.16623	Up	COL3A1
2.157614	9.930151	17.88617	1.18E−47	4.86E−45	97.7363	Up	CTHRC1
3.561535	5.093026	17.76968	3.07E−47	1.20E−44	96.78941	Up	SPRR1A
3.745945	5.457904	17.76204	3.27E−47	1.21E−44	96.72732	Up	GPR87
− 3.12637	5.905882	− 16.8918	4.13E−44	1.05E−41	89.63863	Down	SERTM1
3.032648	6.806622	16.80599	8.37E−44	1.95E−41	88.93822	Up	CHRDL2
− 2.07597	11.46303	− 16.7484	1.34E−43	3.00E−41	88.46867	Down	FIGF
3.160266	12.05447	16.70106	1.98E−43	4.24E−41	88.08196	Up	MMP7
4.805732	9.414086	16.56306	6.16E−43	1.26E−40	86.95575	Up	MMP1
2.037572	6.383056	16.42577	1.91E−42	3.49E−40	85.83511	Up	P4HA3
− 2.19976	3.898955	− 16.41	2.17E−42	3.92E−40	85.70644	Down	DAO
2.226205	4.358469	16.16479	1.63E−41	2.58E−39	83.70469	Up	CPNE4
− 3.76646	10.06016	− 16.1418	1.97E−41	3.08E−39	83.51712	Down	ITLN2
− 2.90756	5.198867	− 16.0732	3.46E−41	5.20E−39	82.95688	Down	SLC5A9
− 2.2071	6.223977	− 16.0238	5.20E−41	7.51E−39	82.55381	Down	MATN3
2.47714	7.467569	15.99126	6.79E−41	9.63E−39	82.28814	Up	MMP11
− 2.47715	14.60113	− 15.9736	7.85E−41	1.10E−38	82.1439	Down	AGER
2.564527	6.131047	15.88976	1.56E−40	2.12E−38	81.45978	Up	GJB2
− 2.24312	6.256766	− 15.7125	6.72E−40	8.43E−38	80.01307	Down	DPP6
2.684995	7.423303	15.60143	1.67E−39	1.97E−37	79.10736	Up	SCRG1
− 2.3598	6.071668	− 15.547	2.62E−39	2.96E−37	78.66324	Down	CCK
− 2.01366	5.110895	− 15.4987	3.89E−39	4.25E−37	78.26978	Down	RGS9BP
− 2.53328	7.572299	− 15.3963	9.02E−39	9.31E−37	77.43515	Down	BTNL9
− 3.14081	9.246772	− 15.3426	1.40E−38	1.37E−36	76.99693	Down	CA4
− 2.01677	5.500521	− 15.1519	6.70E−38	6.28E−36	75.44365	Down	GRIA1
2.63809	9.547163	14.99355	2.46E−37	2.17E−35	74.15484	Up	SFRP2
2.177063	9.87252	14.99053	2.52E−37	2.21E−35	74.13025	Up	CILP
− 2.51131	6.897446	− 14.8662	6.97E−37	5.82E−35	73.11921	Down	ARC
2.977688	4.686335	14.70242	2.67E−36	2.05E−34	71.78832	Up	GREM1
− 2.22583	10.08823	− 14.5432	9.80E−36	6.79E−34	70.49599	Down	CSRNP1
3.273104	5.371113	14.53062	1.09E−35	7.43E−34	70.39393	Up	UGT1A6
2.048878	8.000404	14.40344	3.07E−35	1.99E−33	69.36293	Up	PNOC
2.458664	6.380619	14.35154	4.69E−35	2.93E−33	68.94254	Up	KIAA0125
2.314409	11.53685	14.24544	1.11E−34	6.58E−33	68.08363	Up	POU2AF1
2.810528	11.71609	14.16438	2.16E−34	1.23E−32	67.42805	Up	KRT15
2.473771	8.42905	14.02724	6.59E−34	3.53E−32	66.32004	Up	FCRL5
2.27024	11.15122	13.96948	1.05E−33	5.57E−32	65.85389	Up	MZB1
− 2.57245	5.211454	− 13.8693	2.38E−33	1.22E−31	65.04635	Down	GRM8
2.231172	9.119239	13.8484	2.82E−33	1.41E−31	64.8776	Up	TNFRSF17
2.756707	4.877502	13.78906	4.56E−33	2.23E−31	64.39965	Up	PCSK1
− 2.62411	9.695784	− 13.7279	7.49E−33	3.54E−31	63.90757	Down	RTKN2
2.120844	5.910121	13.62829	1.68E−32	7.30E−31	63.10645	Up	MEOX1
− 2.4414	9.822001	− 13.5846	2.39E−32	1.02E−30	62.75567	Down	VIPR1
2.240894	4.784055	13.50426	4.58E−32	1.90E−30	62.11065	Up	OGDHL
3.053751	8.157115	13.50421	4.58E−32	1.90E−30	62.11025	Up	PLA2G2A
2.129504	4.529648	13.49352	5.00E−32	2.04E−30	62.02447	Up	HS6ST3
4.099913	8.125221	13.44575	7.35E−32	2.92E−30	61.6414	Up	KRT5
2.02468	15.36297	13.43145	8.25E−32	3.26E−30	61.52684	Up	IGLL1
2.957404	8.32699	13.37414	1.31E−31	5.08E−30	61.06769	Up	SLN
2.025418	6.606265	13.32849	1.89E−31	7.24E−30	60.7023	Up	JSRP1
2.955387	8.370847	13.14	8.65E−31	3.07E−29	59.19618	Up	KRT17
2.469841	4.782865	13.13835	8.76E−31	3.11E−29	59.183	Up	ADAMTS16
2.665355	4.140607	13.11133	1.09E−30	3.82E−29	58.96753	Up	DSC3
2.047263	6.544204	12.95095	3.95E−30	1.29E−28	57.69017	Up	SIX4
2.162986	7.005848	12.79213	1.41E−29	4.33E−28	56.42886	Up	KRT13
− 3.82993	6.855422	− 12.7626	1.78E−29	5.42E−28	56.19501	Down	SLC6A4
2.050099	3.420621	12.71757	2.55E−29	7.62E−28	55.83794	Up	GLB1L3
3.327225	10.25723	12.398	3.25E−28	8.42E−27	53.31524	Up	SPP1
2.365788	5.364215	12.3891	3.49E−28	9.01E−27	53.24522	Up	NELL1
2.233526	8.432288	12.25826	9.84E−28	2.44E−26	52.21743	Up	B3GNT3
3.18778	4.471553	11.73307	6.11E−26	1.23E−24	48.12406	Up	KRT6C
3.074268	6.14006	11.57827	2.04E−25	3.88E−24	46.92814	Up	CXCL13
2.28213	8.169303	11.5444	2.66E−25	5.00E−24	46.6672	Up	TNS4
2.552859	4.618913	11.52658	3.05E−25	5.69E−24	46.52998	Up	CYP24A1
2.211397	9.229355	11.52258	3.15E−25	5.83E−24	46.49923	Up	LGALS7
− 2.62597	6.240672	− 11.4101	7.54E−25	1.34E−23	45.63487	Down	HTR3C
− 2.51063	7.189042	− 11.27	2.22E−24	3.74E−23	44.56281	Down	IL1RL1
− 2.18817	3.733392	− 11.1975	3.89E−24	6.42E−23	44.00948	Down	HMGCS2
2.191917	9.204335	11.16278	5.08E−24	8.26E−23	43.74504	Up	SIX1
− 3.09117	10.64484	− 11.15	5.60E−24	9.07E−23	43.64818	Down	FOSB
2.105424	5.55814	10.97058	2.21E−23	3.35E−22	42.2868	Up	FCRLA
3.006776	5.260356	10.94717	2.65E−23	3.95E−22	42.1098	Up	MMP10
2.358111	5.915948	10.92789	3.07E−23	4.56E−22	41.96412	Up	VSIG1
− 2.33134	4.765235	− 10.7303	1.38E−22	1.94E−21	40.47703	Down	ANKRD1
2.142038	8.747571	10.61317	3.34E−22	4.54E−21	39.60094	Up	LCN2
− 3.46187	6.368909	− 10.5444	5.60E−22	7.46E−21	39.08815	Down	CSF3
− 2.07382	12.65793	− 10.5361	5.96E−22	7.93E−21	39.02614	Down	FCN3
2.170145	4.032137	10.39294	1.74E−21	2.20E−20	37.96391	Up	GJB5
2.367125	5.052865	10.32556	2.88E−21	3.58E−20	37.4659	Up	CCL7
2.482971	5.13548	10.24086	5.42E−21	6.52E−20	36.84194	Up	TTR
2.042244	6.607493	10.10804	1.45E−20	1.67E−19	35.86794	Up	LGSN
− 2.00184	5.891826	− 10.0318	2.55E−20	2.86E−19	35.31159	Down	ESM1
− 2.45081	3.630636	− 9.81136	1.28E−19	1.35E−18	33.71326	Down	CT45A1
2.731854	6.087442	9.800989	1.38E−19	1.45E−18	33.63845	Up	C4orf7
2.314047	8.881864	9.489329	1.32E−18	1.24E−17	31.40884	Up	SOX2
− 2.14701	4.465319	− 9.47809	1.43E−18	1.34E−17	31.3291	Down	FAM71A
2.393	6.242972	9.406946	2.38E−18	2.19E−17	30.82539	Up	VTCN1
2.153101	7.83975	9.367191	3.16E−18	2.87E−17	30.54476	Up	RHOV
2.167415	4.486344	9.272752	6.20E−18	5.49E−17	29.88051	Up	CLCA2
2.043337	9.418979	9.156337	1.41E−17	1.21E−16	29.06646	Up	MUC4
2.375417	5.44786	9.103523	2.05E−17	1.74E−16	28.69891	Up	KLK12
3.702802	10.68933	9.001632	4.19E−17	3.42E−16	27.993	Up	BPIFB1
2.28831	5.361617	8.85303	1.18E−16	9.28E−16	26.97114	Up	CXCL6
2.101133	5.911965	8.786643	1.87E−16	1.44E−15	26.51762	Up	ATP12A
2.155802	8.151967	8.776072	2.01E−16	1.55E−15	26.44557	Up	CCNO
2.039754	9.860517	8.654708	4.63E−16	3.42E−15	25.62194	Up	GSTA5
2.296708	7.418001	8.454847	1.81E−15	1.25E−14	24.27972	Up	MUC5B
2.387684	7.938451	8.354771	3.55E−15	2.39E−14	23.61443	Up	C10orf81
2.152617	6.603537	8.293087	5.37E−15	3.56E−14	23.20668	Up	SRD5A2
2.254316	5.652982	8.087896	2.10E−14	1.31E−13	21.86327	Up	MUC16
3.0035	6.116775	7.994348	3.89E−14	2.36E−13	21.25755	Up	SERPINB3
2.920143	5.986109	7.948002	5.26E−14	3.14E−13	20.95907	Up	SERPINB4
2.467293	8.119221	7.861204	9.26E−14	5.38E−13	20.40295	Up	PIP
2.301349	7.277284	7.813318	1.26E−13	7.25E−13	20.09778	Up	TSPAN19
2.126246	10.9766	7.618692	4.41E−13	2.38E−12	18.86961	Up	TMEM190
2.063954	7.274754	7.426121	1.49E−12	7.59E−12	17.67409	Up	ZBBX
3.187369	7.263725	7.35898	2.26E−12	1.14E−11	17.26199	Up	MSMB
2.878548	9.958996	4.790707	2.75E−06	7.11E−06	3.614207	Up	RPS4Y1
2.805762	9.788328	4.671305	4.74E−06	1.19E−05	3.092936	Up	RPS4Y2

### Establishment of IPF classification model with artificial neural network

We used the top six signatures expression in another dataset of GSE32537 to construct an artificial neural network model using the R software package neuralnet. Taking the four hidden layers as the model parameters, the IPF disease classification model is constructed through the obtained gene weight information. Five-fold cross-validation were performed by the Caret package, pROC package was used to estimate the value of AUC^30^.

### Additional data verification

On three independent datasets (GSE47460, GSE53845 and GSE110147), the validity of the constructed classification scoring model of IPF disease and normal samples was verified. The clinical data of GSE70866 were used to evaluate this potential of this model to indicate the patient prognosis and survival.

### Clinical parameters

**Figure 1:**
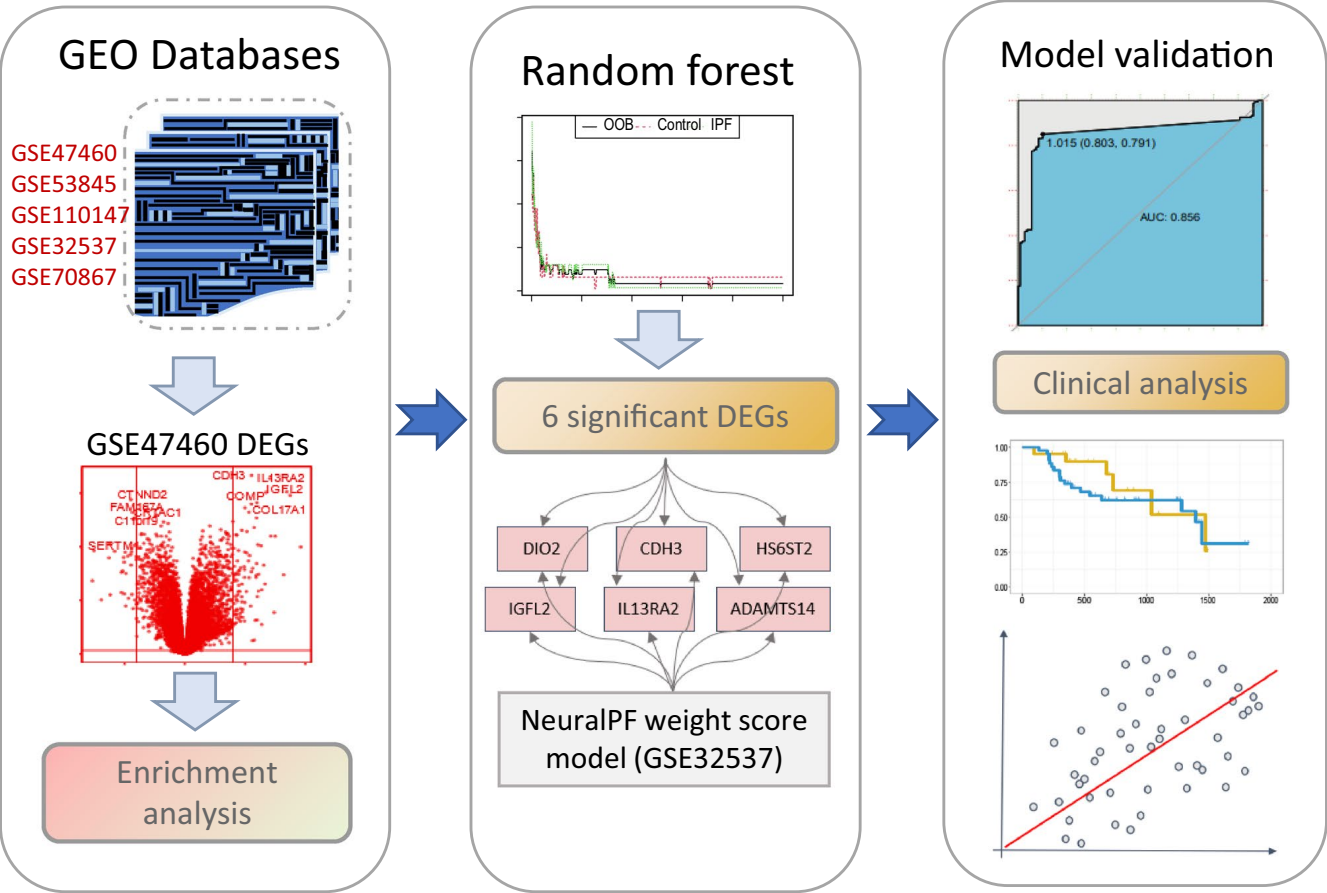
Flow chat.

Clinical straits of IPF patients and healthy control such as the age, gender, pulmonary function tests (PFT) et al. were obtained in GES47460 dataset. The commonly PFT including forced vital capacity (FVC) (% pred.), FVC (post.), forced expiratory volume in 1 s (FEV1) (%pred.), FEV1(post.), and diffusing capacity of the lung for carbon monoxide (DLCO) (%pred.)^31^ were combined into a single "meta" lung function indicator by R package factoextra. FVC(post) and FEV1(post) refer to the post-bronchodilator forced vital capacity (FVC) and forced expiratory volume in one second (FEV1). The design of this study including the main four-step process was represented by a flow chat (Fig. 1).

### Statistical analysis

The R package limma is used for differential expression analysis. To fit the expression data to a linear model and perform empirical Bayes moderation to shrink the standard errors and increase the accuracy of the results. Visualize the results using limma’s built-in plotting functions or by exporting the data and using ggplot2 for visualization. To remove batch effects using the Combat package, the combat() function to adjust for batch effects by specifying the variables that contain the batch information and the variables to be adjusted. OS time and cause of death were obtained and matched to respective patients from the supplemental clinical data available from the GSE70866. Survival time was measured in days starting at diagnosis and ending on the patient’s death or end of the follow-up period. Kaplan–Meier method was used to estimate overall patient survival by genes expression. The high—and low-risk groups were differentiated according to the expression value, with each group containing at least one third of the total sample. The log–rank and Wilcoxon tests were used to compare survival distributions. Correlations were calculated using Spearman’s rank correlation (presented as Spearman rho). The resulting coefficient will range from − 1 to 1, where  − 1 indicates a perfect inverse relationship, 0 indicates no relationship, and 1 indicates a perfect direct relationship.

## Results

### Significantly alteration of the genes and enriched signal pathway in IPF

**Figure 2:**
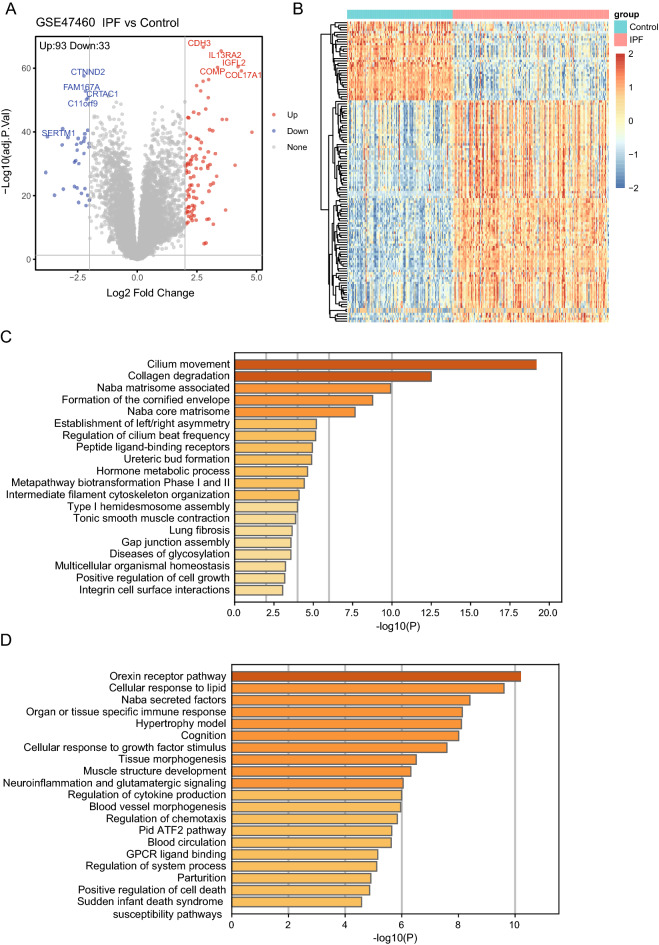
Differential gene expression analysis in IPF. (**A**) Volcano plot of differential expression analysis results. The abscissa is log2Fold Change and the ordinate is –log10 (adj.*P* value). The upper right part has a adj.*P* value less than 0.05 and a fold change greater than 2, indicating significant DEGs with higher expression levels. The upper left part has a adj.*P* value less than 0.05 and a fold change less than − 2, indicating significant DEGs with reduced expression. The gray dots represent the remaining stable genes. (**B**) Heatmap of DEGs. The colors in the graph from red to blue indicate high to low expression. On the upper part of the heatmap, the red band indicates the disease samples and the blue band indicates the normal samples. C-D. Matescape toll function enrichment results bar graph. The x-axis represents −log10(adj *P*) values and the y-axis represents enriched pathways. Pathways with Log10(*P* value) of > 2.5 are marked and shown in the figure. (**C**) shows a bar graph of the enriched pathways that were significantly up-regulated in IPF patients compared to healthy controls. (**D**) shows a bar graph of the enrichment pathway results that were significantly downregulated in IPF patients compared to healthy controls.

The design of this study including the main four-step process was represented by a flow chat (Fig. 1). Totally 213 subjects in the GSE47460 dataset, including 91 healthy control and 122 IPF patients, the Bayesian test in the limma package were used to identify DEGs between normal and IPF samples. One hundred and twenty-six significantly differentially expressed proteins (DEPs) (adj.P.Val < 0.05 and a differential expression ratio [IPF/N] > 2 or < 0.5), including 93 up-regulated genes and 33 down-regulated genes were identified and heatmap in Fig. 2A, B. Then the metascape tool was used for pathway enrichment analysis of 126 important DEGs, the thresholds set at a -Log_10_ (*P* value) of > 2.5. Of the DEGs upregulated in patients with IPF were enriched in pathways associated with collagen degradation, NABA CORE MATRISOME and lung fibrosis (Fig. 2C), whereas DEGs those involved in cellular response to lipid, regulation of cytokine, positive regulation of cell death (Fig. 2D). These pathways were combined with GO database, KEGG database, Reactome database and Wikipathway database.

### Random forest classifies the DEGs between IPF and healthy control

**Figure 3:**
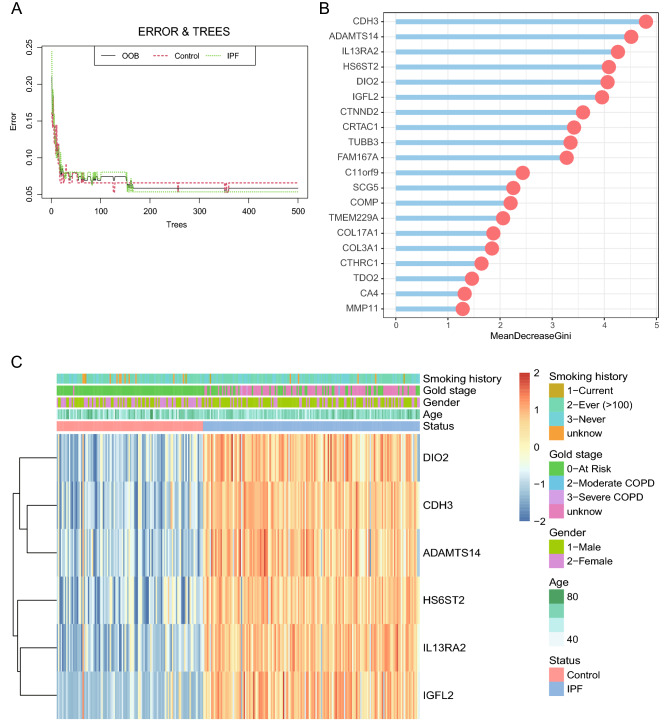
Random Forest screening for DEGs. (**A**) The effect of the number of decision trees on the error rate. The x-axis represents the number of decision trees, and the y-axis represents the error rate. When the number of decision trees is about 500, the error rate is relatively stable. (**B**) Results of the Gini coefficient method in the random forest classifier. The x-axis represents the importance index, and the y-axis represents the genetic variables. Rank and display the top 20 genes of importance coefficient. (**C**) The unsupervised clustering heatmap shows the hierarchical clustering results generated from six significant genes generated by a random forest in GSE47460. On the upper part of the heatmap, the red band in the status module represents normal samples, and the blue band represents disease samples; the color in the age module gradually changes from white to green, representing the increasing age of the sample; the light green band in the gender module represents male samples, the purple strip represents female samples; the green strip in the gold stage module means AT Risk, the green strip means Moderate COPD; the purple strip means Severe COPD; the rose-red strip means unknown; the yellow strip in the smoking history module means the current still Smoking; green strips have ever smoked; blue strips have never smoked; orange strips are unknown.

The 126 DEGs were further classified by random forest classifier. In order to further obtain a model with stable error, appropriate parameters are selected by changing the number of decision trees, and finally 500 trees are set as the optimal parameters of the model (Fig. 3A). Nine was determined as the parameter of variable number, the importance of features by calculating the purity of nodes through Gini coefficient method were computed, the top 20 potential indicators were showed in Fig. 3B. Next, DEGs with importance greater than 4 or equal to 4 were screened for further analysis. which are CDH3, ADAMTS14, IL13RA2, HS6ST2, DIO2 and IGFL2 sequentially (Fig. 3B). The association between the top six genes’ expression with the age, gender, smoking history and disease stage, and status were heatmap in Fig. 3C by k-means unsupervised cluster, which indicated that the genes panel can be used to distinguish IPF patients from the control samples. Although older age and male increased susceptibility to IPF^32^, there was no significant correlation between the expression of the six genes and age or sex (Fig. S1). This suggests that the six genes are not affected by these factors in distinguishing between normal and IPF.

### Construction of the artificial neural network model

**Figure 4:**
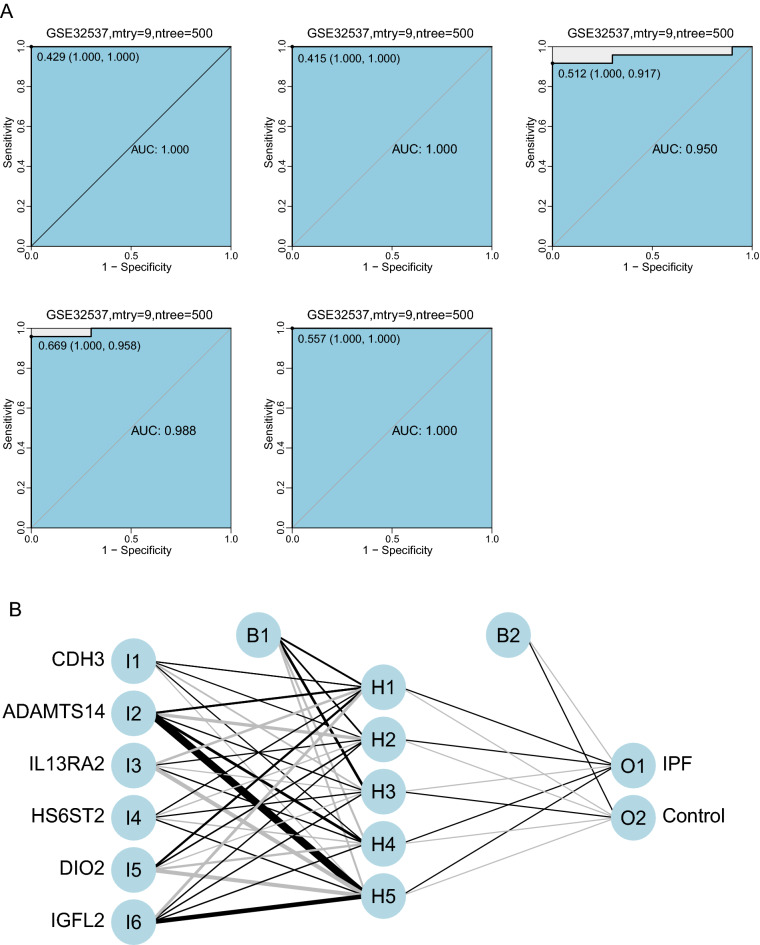
Construction of the artificial neural network model. (**A**) Verification of the ROC curve results by the five-time cross-validation model in GSE32537. The points marked on the ROC curve are the optimal threshold points, and the values in parentheses represent sensitivity and specificity. The AUC value is the area under the ROC curve. (**B**) Results of neural network visualization.

The convolutional neural network model was constructed using another dataset GSE32537 by the neuralnet package. Before training the neural network model, we need to set important parameters, especially the number of hidden layers and the number of neurons. There were no fixed rules for the setting of these two values, which relied more on experience and constant attempt. After many tests on the number of hidden layers, we found that when the number of hidden layers was 5, the training effect of the model was the best. Six neurons were finally set as model parameters based on the size of the input layer, commonly two-thirds of the input size was recommended. In order to further strengthen the stability of the neural network model, the GSE32537 dataset was randomly divided into the training set and the validation set, the fivefold cross-validation method was used for 5 iterations of optimization. The more important DEGs and their corresponding weight coefficients were learned from the training set. The classification effect of the scoring model was proved by the validation on other datasets, and the classification accuracy of the neural network model on the verification set was recorded each time. The receiver operating characteristic (ROC) curve is used to evaluate the classification performance of the model. The fivefold cross-validation results show that the AUC value of the area under the ROC curve is close to 1 (average AUC ≈ 0.99) (Fig. 4A), which indicating that the classification accuracy of this convolutional neural network model was high.

The training of the whole neural network model was performed in 28,730 steps, In the connection weights between neurons of the network, the positive weights were connected by black lines, the negative weights were connected by gray lines, and the thickness of the lines reflected the value of the weight. The termination condition of neural network training was the absolute partial derivative of the error function was less than 0.01(almost reached threshold), and the output result of the entire training process shown in Fig. 4B.

### Model accuracy verification

**Figure 5:**
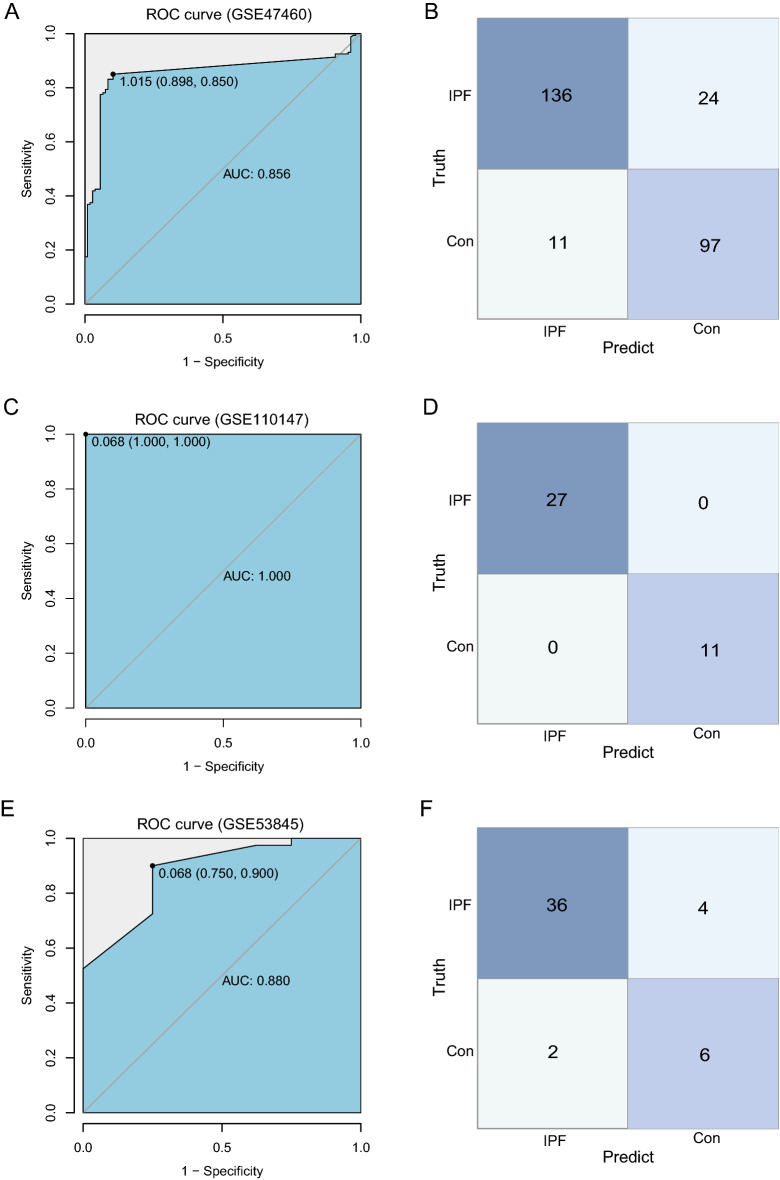
Model accuracy verification. (**A**) Verification of the ROC curve results in GSE47460. The points marked on the ROC curve are the optimal threshold points, and the values in parentheses represent sensitivity and specificity. The AUC value is the area under the ROC curve. (**B**) GSE47460 confusion matrix result. The x-axis represents the predicted results, and the y-axis represents the actual results. (**C**) Verification of the ROC curve results in GSE110147. The points marked on the ROC curve are the optimal threshold points, and the values in parentheses represent sensitivity and specificity. The AUC value is the area under the ROC curve. (**D**) GSE110147 confusion matrix result. The x-axis represents the predicted results, and the y-axis represents the actual results. (**E**) Verification of the ROC curve results in GSE53845. The points marked on the ROC curve are the optimal threshold points, and the values in parentheses represent sensitivity and specificity. The AUC value is the area under the ROC curve. (**F**) GSE53845 confusion matrix result. The x-axis represents the predicted results, and the y-axis represents the actual results.

The trained neural network model was put into three independent datasets of GSE47460, GSE110147 and GSE53845 for verification. The data in the three datasets were standardized before verification. In GSE47460 dataset, the sensitivity was 90%, the specificity was 85%, and the AUC was 0.856 (Fig. 5A). In GSE110147 dataset, the sensitivity was 100%, the specificity was 100%, and the AUC was 1 (Fig. 5C). In GSE53845, the sensitivity was 75%, the specificity was 90%, and the AUC was 0.880 (Fig. 5E). The confusion matrix results of GSE47460, GSE110147 and GSE53845 are shown in Fig. 5B,D and F respectively. These data demonstrated that the accuracy of this model is reliable.

### Survival analysis

**Figure 6:**
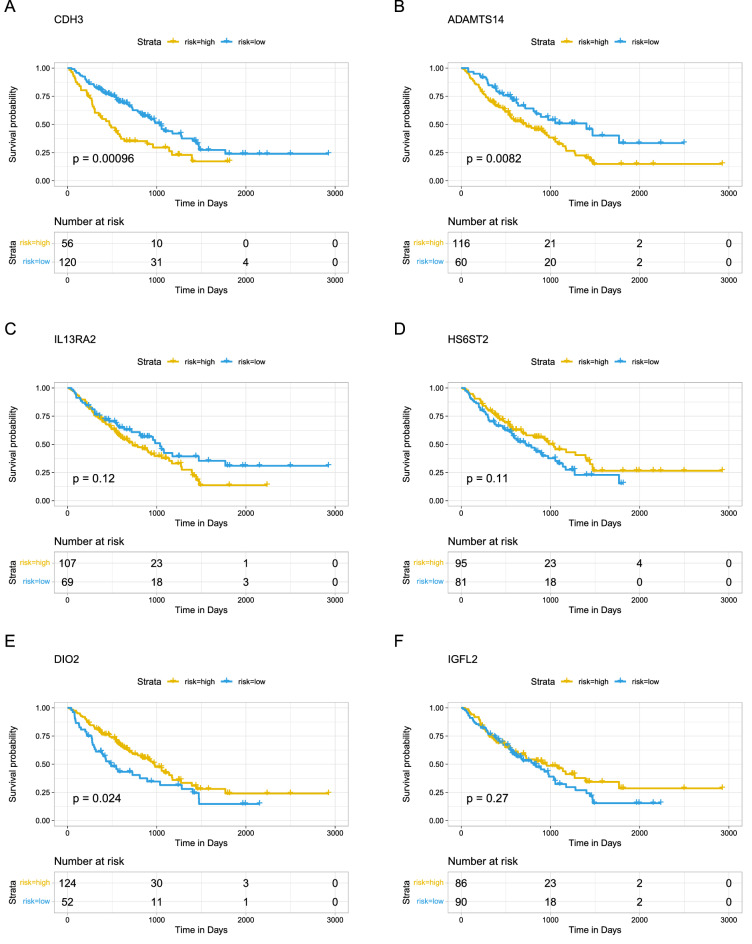
Survival predictive analysis. (**A**) CDH3 as a prognostic factor to evaluate the prognosis and survival status of IPF patients. (**B**) ADAMTS14 as a prognostic factor to evaluate the prognosis and survival status of IPF patients. (**C**) IL13RA2 as a prognostic factor to evaluate the prognosis and survival status of IPF patients. (**D**) HS6ST2 as a prognostic factor to evaluate the prognosis and survival status of IPF patients. (**E**) DIO2 as a prognostic factor to evaluate the prognosis and survival status of IPF patients. (**F**) IGFL2 as a prognostic factor to evaluate the prognosis and survival status of IPF patients. The x-axis represents time and the y-axis represents survival probability. The yellow line represents the high gene expression group, and the blue line represents the gene low expression group. Each point on the curve represents the patient's survival rate at that time point.

To further estimate the prognostic effect of the identified candidate genes in IPF, the complete dataset (GSE70866) of RNA-seq samples (bronchoalveolar lavage fluid) with follow-up comprised 194 specimens from IPF patients (n = 176) and normal controls (n = 18). Cox proportional hazards regression model and Kaplan–Meier method (product-limit method) were used to calculate the correlation between gene expression and survival status. Univariate COX results showed that CDH3 was a potential prognostic marker (HR = 1.3, pvalue = 0.0013, Fig. S2). Of the six signatures, CDH3, ADAMTS14 and DIO2 showed a different significant association with overall survival in IPF (Fig. 6A–F). Patients with high expression of CDH3 and ADAMTS14 had a poor prognosis (Fig. 6A, B), while those with high expression of DIO2 had a good prognosis (Fig. 6E).

### Six signatures correlation analysis with clinical features

**Figure 7:**
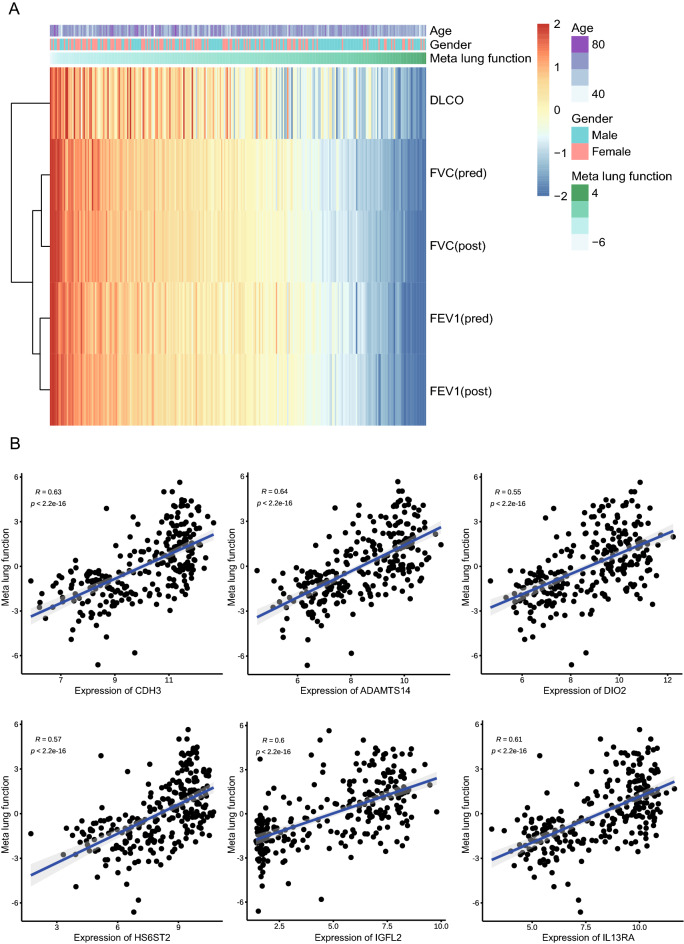
Six signature genes were significantly associated with clinical features. (**A**) The heatmap illustrates the computationally derived meta lung function variable combing multiple lung function parameters. In the upper part of the heatmap, the color in the meta lung function module gradually changes from white to green, representing an increase in the sample meta lung function; the blue bars in the gender module represent male samples, and the red bars represent female samples; the age module The color gradually changes from white to purple, representing the increasing age of the sample. On the right side of the heat map, there are clinical indicators DLCO, FVC (pred), FVC (post), FEV1(pred), and FEV1(post). Pred, predict; Post, post-bronchodilator. (**B**) The scatter plots show the positive correlation of the indicated genes with meta lung function. The x-axis represents gene expression, and the y-axis represents meta lung function.

The dataset (GSE47460) including the RNA-seq counts and clinical data from IPF patients was used to yielded the global correlation network heatmap shown in Fig. 7A. Quantification of multiple combinations of clinical lung function parameters into a single "meta" lung function measure by principal component analysis. Next, we performed linear multivariate regression analysis the mRNA expression levels associated with the meta lung function variable. CDH3, ADAMTS14, IL13RA2, HS6ST2, DIO2 and IGFL2 are positive correlated to lung function with R value at about 0.6 (Fig. 7B).

## Discussion

In this study, we calculated differentially expressed genes (DEGs) related to idiopathic pulmonary fibrosis (IPF), and obtained six important candidate DEGs using a random forest classifier. we used a neural network model to determine the predicted weights of related genes and construct a classification model score for IPF. We then evaluated the classification efficiency of the model score in three independent sample datasets^17^. We found that the AUC efficiency of our model, called neura IPF, was excellent. However, the lack of gene data for IPF in the GEO database meant that the genetic characteristics of IPF were not included in the construction of the diagnostic model, potentially compromising its diagnostic effectiveness for IPF.

Of these six genes, a major function of DIO2 is to convert T4 to active T3 so that TH (Thyroid hormone) can be activated. IPF, diabetic nephropathy, and myocardial infarction have all been associated with a poor prognosis with hypothyroidism^21,33–36^. The expression and activity of DIO2 are increased in the lungs of patients with IPF and are correlated with disease severity. DIO2 mainly localizes to AECs, which are thought to play a central role in the cycle of injury and repair that is characteristic of IPF^21^. DIO2 is significantly upregulated in the fibrotic state, but this upregulation is thought to be protective. This was further confirmed in our prognostic analysis^21^.

Insulin-like growth factors (IGFs) and their binding proteins (IGFBPs) play a critical role in pulmonary fibrosis development and progression^37^. It has previously been shown that IGFBP2 and IGF-like family member 2 (IGFL2) are upregulated in SSc-PF and IPF^38^. IGFL2 is secreted form in the ECM, its expression is also increased in IPF. IGFL2 expression levels were significantly reduced in human skin fibroblasts aged with mitochondrial function, suggesting that mitochondrial physiological processes are associated with IGFL2^38^. IGFL2 play critical roles in cellular energy metabolism and in growth and development, especially prenatal growth. However, there has been no relevant research exploring its role in the pathogenesis of IPF.

Classical cadherins are the principle adhesive proteins at cohesive intercellular junctions and are essential proteins for morphogenesis and tissue homeostasis^39^. P-cadherin is a calcium dependent cell–cell adhesion glycoprotein, which has a crucial role in the conservation of the structural integrity of epithelial tissues. Like other members of the cadherin family, P-cadherin (CDH3) regulates several cellular homeostatic processes that participate in embryonic development and maintain adult tissue architecture, being important for cell differentiation, cell shape, cell polarity, growth, and migration^40^. It is worth noting that DIO2 and IGFL2 also play an important role in growth and development. These three genes play a major role in development, regeneration, morphogenesis and so on. This highlights the prominent role of tissue formation and development in the pathogenesis of fibrosis.

Interleukin (IL)-13 has been shown to play a role in several inflammatory and fibrotic diseases^41^. IL-13 modulates its effector functions via a complex receptor system that includes the IL-4 receptor (R) α, IL-13Rα1, and the IL-13Rα2. IL-13Rα1 binds IL-13 with low affinity, yet, when it forms a complex with IL-4α, it binds with much higher affinity, inducing the effector functions of IL-13. IL-13Rα2 binds IL-13 with high affinity but has a short cytoplasmic tail and has been shown to act as a nonsignaling decoy receptor. Transfection of fibroblasts and epithelial cells with IL-13Rα2 inhibited the IL-13 induction of soluble collagen, TGF-β, and CCL17. Adenoviral overexpression of IL-13Rα2 in the lung reduced bleomycin-induced fibrosis^41^.

Heparan sulfate (HS) proteoglycan is a glycosaminoglycan widely distributed on the surface of animal cells and extracellular matrix, and regulates cell growth, differentiation, adhesion, and migration by interacting with various ligands complement. Compared with normal lung, IPF lung showed significantly increased HS6-O-sulfuration and HS6-O-sulftransferase 1 and 2 (HS6ST1/2) mRNA overexpression. Immunohistochemistry showed that HS6ST2 was specifically expressed in bronchial epithelial cells, including IPF lung honeycomb cyst lining cells^42^. Both IL13RA2 and HS6ST2 highlight the importance of ligand receptor interactions, highlighting that cell–cell interactions in the microenvironment may be a major cause of fibrosis progression.

ADAMTS14 gene encodes a member of the ADAMTS (a disintegrin and metalloproteinase with thrombospondin motif) protein family. As reported by previously studies, the ADAMTS14 gene was discovered to play critical roles in the progress of inflammation and the immune system, through a crosstalk of the TGF-β pathways and mesenchymal cells^43^. ADAMTS14 gene polymorphism was associated with knee osteoarthritis^44^ or the osteoarthritis of the temporomandibular joint in Chinese Han women^45^. But so far, no studies have been able to clarify ADAMTS14's role in pulmonary fibrosis. Like HS6ST2, ADAMTS14 is also mainly present in extracellular matrix, which may suggest that the composition or structure of extracellular matrix is also an important pathological factor that should not be ignored in pulmonary fibrosis.

This model has made significant progress compared to other models in previous studies^37^. This progress is primarily reflected in the use of fewer feature variables, the validation of the model using a large-scale dataset, and its strong predictive performance. There still are some limitations in this study, the sample sizes of the cohorts are still relatively big enoughwhich may not be sufficient to represent the overall population precisely and could affect the generalizability of diagnostic model. Additionally, this diagnostic model is based on preliminary findings and short of the sound experimental verification to support its reliability. As such, given these limitations, this diagnostic model requires further investigation to determine whether it can be used in clinical decision-making.

In conclusion, we constructed an artificial neural network model that demonstrated robust performance across multiple cohorts. We assessed the relationship between each gene of the model and demographic variables. The majority of the genes showed no association with age or gender, but all presented close correlation with clinical features. CDH3, ADAMTS14 and DIO2 were found to be related to prognosis. These results are useful to prioritize targeting these indicators for diagnosis and drug development in future.

## Supplementary Information

Supplementary Information.

## Data Availability

The datasets generated and analysed during the current study are available in the GEO repository, [https://www.ncbi.nlm.nih.gov/geo/].
