# Supplementary material for: Artificial neural network identified the significant genes to distinguish Idiopathic pulmonary fibrosis
Source: Sci Rep. 2023 Jan 21;13:1225. doi: 10.1038/s41598-023-28536-w (PMC9867697; doi:10.1038/s41598-023-28536-w)

Artificial Neural network identified the significant genes to distinguish Idiopathic pulmonary fibrosis

Zhongzheng Li, Shenghui Wang, Huabin Zhao, Peishuo Yan, Hongmei Yuan, Mengxia Zhao, Ruyan Wan, Guoying Yu[*#], Lan Wang[*#]

State Key Laboratory of Cell Differentiation and Regulation, Henan International Joint Laboratory of Pulmonary Fibrosis, Henan Center for Outstanding Overseas Scientists of Pulmonary Fibrosis, College of Life Science, Henan Normal University, Xinxiang, Henan 453007, China

*Corresponding authors. #Contribute equally

Correspondence to:

Guoying Yu, PhD, Email: guoyingyu@htu.edu.cn,

Lan Wang, PhD, Email: wanglan@htu.edu.cn

Tel:86-373-3326340

Henan Normal University, 46 Jianshe Road, Xinxiang, Henan 453007, China

Supplemental figures legends:

Figure S1 Association of DEGs expression with gender and age

A. Boxplot showing the association of the DEGs with gender, x-axis represents genders, y-axis represents gene expression.

B. Scatter plot showing the association of the DEGs with age, x-axis represents age, y-axis represents gene expression.

Figure S2

The forest plot displays the univariate Cox regression analysis results of the risk score and clinical factors with OS.

Figure S1

A

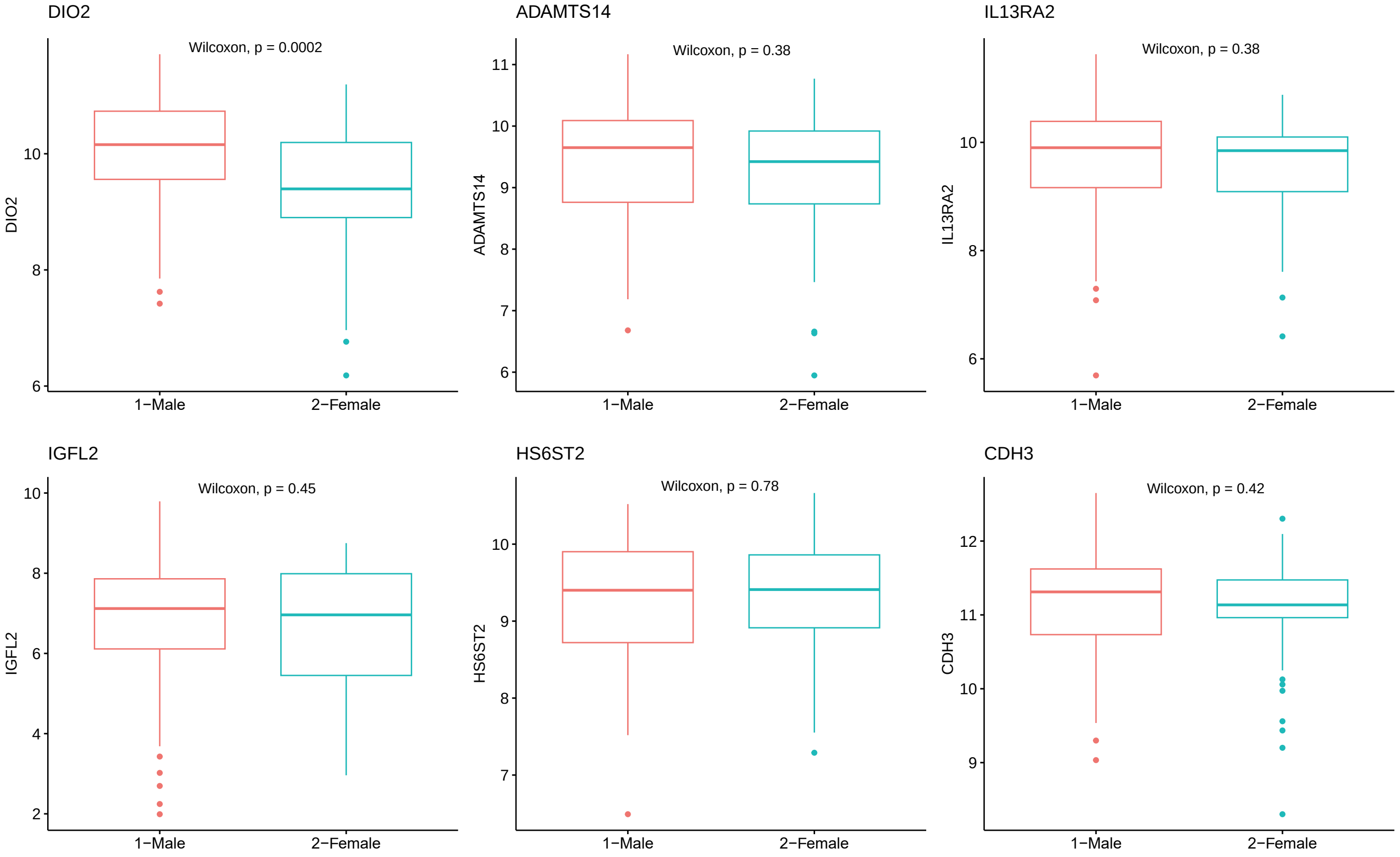
DIO2
Wilcoxon, p = 0.0002
ADAMTS14
Wilcoxon, p = 0.38
IL13RA2
Wilcoxon, p = 0.38
IGFL2
Wilcoxon, p = 0.45
HS6ST2
Wilcoxon, p = 0.78
CDH3
Wilcoxon, p = 0.42
1−Male
2−Female

B

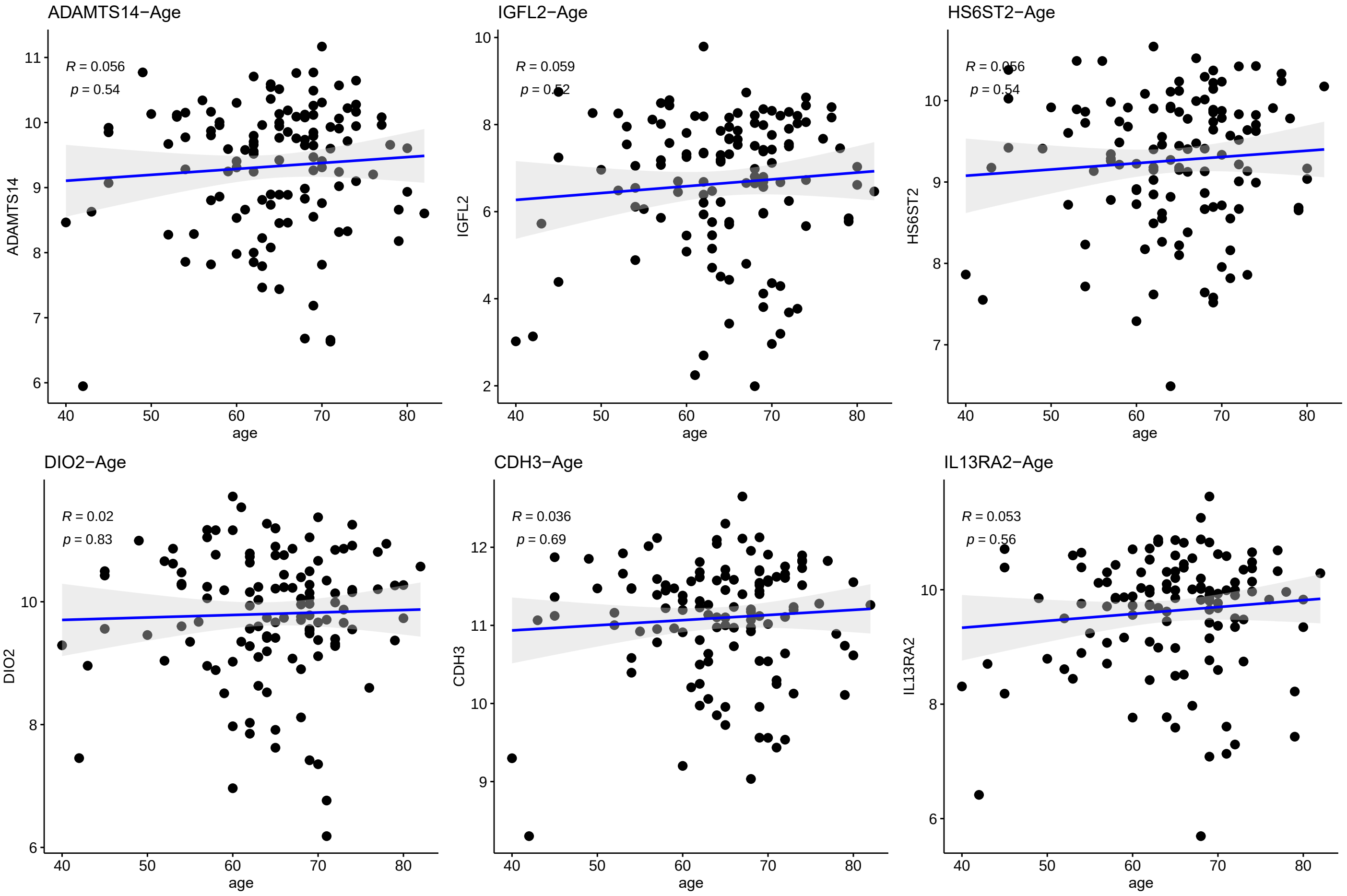
ADAMTS14−Age
R = 0.056
p = 0.54
IGFL2−Age
R = 0.059
p = 0.52
HS6ST2−Age
R = 0.056
p = 0.54
DIO2−Age
R = 0.02
p = 0.83
CDH3−Age
R = 0.036
p = 0.69
IL13RA2−Age
R = 0.053
p = 0.56
age

Figure S2

Gene	Coef	Pvalue	HR(95%CI)
CDH3	0.300	0.0013	1.3 (1.1−1.6)
DIO2	−0.180	0.1100	0.83 (0.67−1)
HS6ST2	−0.088	0.3400	0.92 (0.76−1.1)
ADAMTS14	0.063	0.5100	1.1 (0.88−1.3)
IL13RA2	−0.015	0.9300	0.99 (0.71−1.4)
IGFL2	0.001	0.9900	1 (0.84−1.2)

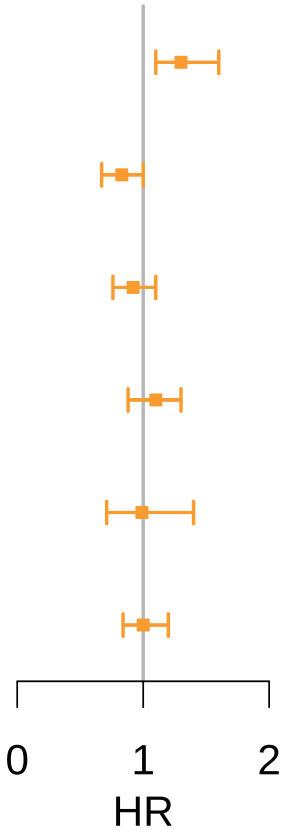

Supplement: Supplementary file 1 — Supplementary Information. [file 41598_2023_28536_MOESM1_ESM.pdf]
